# Functional Needs Assessment Tool for estimating population-level functional difficulties and the need for services and assistive products: pilot testing in Western Uganda

**DOI:** 10.3389/fresc.2026.1766661

**Published:** 2026-07-21

**Authors:** Dorothy Boggs, Annet Birabwa, Suzanne Adkins, Oluwarantimi Atijosan-Ayodele, Sahan Bulathwela, Catherine de Cates, Thomas Doggett, Allen Foster, Hannah Kuper, Cathy Holloway, Joseph Mugisha Okello, Sarah Polack, FNAT team

**Affiliations:** 1 International Centre for Evidence in Disability, London School of Hygiene & Tropical Medicine, London, United Kingdom; 2 Medical Research Council/Uganda Virus Research Institute and London School of Hygiene & Tropical Medicine Uganda Research Unit, Entebbe, Uganda; 3 King’s College Hospital NHS Foundation Trust, London, United Kingdom; 4 Department of Computer Science, Global Disability Innovation Hub, University College London, London, United Kingdom; 5 Centre for Artificial Intelligence, University College London, London, United Kingdom; 6 Department of Otolaryngology, Lister Hospital, East and North Hertfordshire NHS Trust, Stevenage, United Kingdom; 7 Cambridge Global Health Partnership, Cambridge, United Kingdom; 8 School of Medicine, Anglia Ruskin University, Chelmsford, United Kingdom; 9 International Centre for Eye Health, London School of Hygiene & Tropical Medicine, London, United Kingdom

**Keywords:** assistive products, functioning, impairment, rehabilitation, screening, services, surveys, Uganda

## Abstract

**Background:**

Reliable data on population-level need for rehabilitation services and assistive products (AP) across different settings are lacking. The Functional Needs Assessment Tool (FNAT) is a new survey method for estimating the prevalence of functional difficulties/impairment and the need for services and AP. It combines self-report, clinical impairment and functional assessments across seven domains: vision, hearing, mobility, communication, cognition, self-care, and mental health. Data are collected using a bespoke mobile application and web-based platform. The FNAT methodology is presented in the first paper in this series.

**Objective:**

The FNAT was pilot-tested in Kalungu District, Uganda, between March and July 2023 to assess the feasibility of the methodology and the functionality of the mobile data collection application.

**Methods:**

Two pilot studies were conducted across 18 communities/villages, including 305 (pilot 1) and 550 (pilot 2) participants aged 2 years and older. Participants were identified using a two-stage cluster sampling method and underwent assessments using standardised tools to evaluate functional difficulties/impairments and the need for services and AP.

**Results:**

The pilot studies identified several key learnings, such as the need for questionnaire revisions and improvements to the application programming. It confirmed the feasibility of completing the two-stage assessments over 2 days with a cluster size of 45 participants (participants aged 2 years and older) while including visual acuity screening for all participants.

**Discussion:**

The FNAT is a feasible approach for estimating the prevalence of functional difficulties and the need for and coverage of related services and AP, with lessons learned for future development, and should be tested at scale. The field-test study is presented in the final paper in this series.

## 1 Introduction

An estimated 2.6 billion people globally experience difficulties with functioning and would benefit from rehabilitation (1, 2), and more than 2.5 billion people need assistive technology (AT) (3). Rehabilitation and AT can be fundamental for facilitating independence and participation for people with functioning difficulties and improving their well-being (4). Diverse groups, including people with disabilities, older people, and people with chronic conditions, can benefit from these services (5, 6). However, the unmet need for rehabilitation and AT remains high and continues to increase (3, 6). Currently, there is a lack of reliable data on population need to inform policy development and service provision. Such data are particularly needed in low- and middle-income country (LMIC) settings, where access to both rehabilitation and assistive products (AP) is often limited (7, 8).

### 1.1 Overview of the Functional Needs Assessment Tool

In response to these gaps and challenges, in collaboration with the AT2030 consortium (9) and the Computer Science Department at the University College London (UCL), the International Centre for Evidence in Disability (ICED) at the London School of Hygiene and Tropical Medicine (LSHTM) developed the Functional Needs Assessment Tool (FNAT), a new multi-domain survey methodology and bespoke tablet-based mobile data collection application.

FNAT aims to estimate the (i) prevalence of functional difficulties/impairments and (ii) the need for related health and rehabilitation services and AP in people aged 2 years and older. The survey uses a two-stage cluster random sampling methodology to identify participants and a two-stage assessment process: an initial screening (day 1), followed by clinician-led assessments (day 2) for those who screen positive. The tool includes a combination of self-report, clinical impairment and functional assessments to identify service and AP needs across seven domains: vision, hearing, mobility, communication, cognition, self-care, and mental health.

The survey method and the bespoke tablet-based mobile data collection application (mFNAT) needed to be pilot-tested. The key steps involved in the development of the FNAT modular tool are described in the first paper in this series (10).

### 1.2 FNAT pilot test in Uganda

In this paper, we present the methods and results of a pilot test of the survey and tablet-based mobile application in Kalungu District, Uganda. National estimates suggest that 11%–13% of the population experience at least some functional difficulty (11, 12) and that the need for rehabilitation and AT is likely to increase, driven by rising life expectancy as well as increases rates of non-communicable diseases and injuries. In Kalungu, testing of this population-based tool for collecting district-level data is important for informing future research and service development.

The specific objectives were to assess (1) the acceptability and comprehension of the questionnaires; (2) the functionality of the FNAT tablet-based mobile application and web-based platform; (3) the overall feasibility of the two-stage assessment method; (4) the number of participants that could be examined per day; and (5) the feasibility of including vision and hearing screening for all participants.

## 2 Materials and methods

FNAT was pilot-tested in Kalungu District between March and July 2023.

Pilot testing consisted of three components:

- Clinic-based testing of questionnaires with 2–5 participants per domain to assess acceptability, understanding, translation, and administration time (March 2023, Objective 1).
- Pilot 1: testing the survey in six clusters (17 April to 16 May 2023, Objectives 1 and 2).
- Pilot 2: testing the survey in 12 clusters (25 May to 4 July 2023, Objectives 2–5).

Feedback was obtained from the survey teams on the questionnaire (e.g., wording and comprehension), translation, and survey processes through daily debriefings conducted during training and throughout the pilot studies. The process was iterative; e.g., learnings from pilot 1 were incorporated into pilot 2, and data were collected using the mFNAT platform. In addition, a study advisory committee was established prior to pilot testing, comprising local and national stakeholders, including service providers and policymakers, to guide the research throughout the study. In this paper, “service need” refers to need for health and rehabilitation services.

### 2.1 Study setting and population

Kalungu District is located in rural southwestern Uganda. The district comprises five subcounties, 33 administrative parishes, and 371 villages. Agriculture is the main economic activity in the area. Within the district, there are two level IV health centres (with a doctor and other health workers), and each subcounty has a public level III health centre that provides both curative and preventive services. In addition, there is a private district-level hospital. Most people needing rehabilitation services are referred to Masaka Regional Referral hospital, which neighbours Kalungu District. The survey was coordinated from Kyamulibwa Subcounty, where the MRC/UVRI and LSHTM Uganda Research Unit has a station.

FNAT was developed to include people aged 2 years and older across seven functional domains. However, restricting to an older population, where the prevalence of functional difficulties and the need for AP are highest, and/or limiting the number of domains assessed would require a smaller sample size and therefore reduce survey duration. The choice of age groups and functional domains included will depend on resources, time, and information needs. In this study, we pilot-tested all seven domains of FNAT in the 2 years and older age group and also reviewed data for those aged 40 years and older, based on contextual resources, time, and information needs.

### 2.2 Survey team and training

There were two survey teams, each comprising three interviewers, one ophthalmic clinical officer (OCO), one ENT clinical officer (ENT CO), one physiotherapist, and one occupational therapist (OT). The survey teams were composed of trained Ugandan professionals, fluent in the local language Luganda, and underwent 10 days of training at the Kyamulibwa MRC/UVRI and LSHTM Uganda Research Unit. The training covered survey aims, methods, referrals, ethical considerations, disability awareness, quality standards, and domain-specific training. Three survey teams were originally planned, but this was not possible due to recruitment issues.

### 2.3 Sampling

FNAT uses the same two-stage cluster random sampling method (13) as other impairment-based surveys. Clusters (typically census enumeration units) are selected as primary sampling units using probability proportionate to size sampling. People within clusters are selected using a form of compact segment sampling, whereby a fixed number of people e.g. 40 to include per cluster is decided from the outset (14). Using community maps (15), each cluster is divided into segments, with each segment containing approximately the target number of participants, e.g., 40. A random edge starting point is selected, and households are visited door-to-door until the target number of participants is reached. We tested different cluster sizes to determine the number of people that could feasibly be assessed in a single day for both stage-one and stage-two assessments.

### 2.4 Data collection

#### 2.4.1 Overview and coordination

For each cluster, data collection lasted 2 days. Day 1, conducted by interviewers, included enumeration, questions on AP awareness and use, and first-stage screening. Day 2 involved clinician-led second-stage assessments for participants who screened positive on Day 1. For this multi-domain survey, this approach was considered logistically more feasible than completing first- and second-stage assessments on the same day (16). In addition, the tablet-based mobile application and web-based dashboard were designed to list people who screened positive on Day 1, which was vital for coordinating and completing stage-two assessments; however, this function required internet connectivity and hence could not be completed within clusters in a single day. Therefore, the two-day approach also facilitated coordination and scheduling of clinician visits.

#### 2.4.2 Enumeration

A Village Local Council member, or their representative who was familiar with the area, accompanied the survey teams. Informed consent was obtained from the household head. A household roster was completed to record the name, age, sex, education level, occupation, marital status, and contact details of each household member aged 2 years and older. Data on household-level socio-economic indicators were collected using the Equity Tool (17). All household members were invited to participate in the study, and informed consent was obtained from those who agreed to participate.

#### 2.4.3 First-stage assessments

##### 2.4.3.1 Self-reported tools

Table 1 presents the self-reported screening tools administered to all participants. For more details, refer to the first development paper in this series (10).

**Table 1:** FNAT pilot test 2 two-stage functional difficulty/impairment screening and assessment tools/questions by domain and age groups.

Two stage tools	Children		Adults
	2–4 years old	5–17 years old	18 years and older
Stage 1 domains			
Multiple functional domains^a^	UNICEF/WG Child Functioning Module (CFM) (25)	UNICEF/WG CFM (25)	WG Extended Set (ES) of Questions (25)
Mobility	Rapid Assessment Musculoskeletal Impairment (RAM) six screening questions (26, 27)		
Communication	Communication Disability Screening Tool (CDST) first three screening questions (28, 29)		
Self-care: incontinence	–	Adapted Vanuatu Women, Water and Disability Study four screening questions (30)	
AP awareness, use, and access^b^	Modified Rapid Assistive Technology Assessment (rATA) (22)		
Vision^c^	–	Age 5 years and older: Peek Acuity distance visual acuity (VA) test (18)	Age 40 years and older: Peek Near Vision near VA screening (19)
Stage 2 domains			
Vision	Fix-and-follow methods (<3 years) Counting fingers (3–4 years)	Modified RAAB (31) using Peek Acuity (23)	Modified RAAB (31) using Peek Acuity (18, 23) Peek Near Vision screening (19)
Hearing	Otoacoustic Emissions (OAE) test or reported question asked to a relative/neighbour^d^	Modified RAHL (32) using hearTest (33)	
Mobility	Updated full RAM assessment (26, 27)		
Communication	CDST last two screening questions (28, 29)		
Cognition	UNICEF/WG CFM understanding, learning, playing, and behaviour	UNICEF/WG CFM learning, remembering, concentrating, accepting changes, controlling behaviour, or socialising	Age 18–59 years: WGES remembering and/or concentrating Age 60 years and older: modified Oxford Cognitive Screen-Plus (OCS-Plus) (34, 35)
Self-care	–	UNICEF/WG CFM self-care	Barthels Index (BI) (36) Adapted Vanuatu Women, Water and Disability Study six screening questions (30)
Mental health	–	UNICEF/WG CFM depression and anxiety	Patient Health Questionnaire (PHQ)-9 (37) Generalised Anxiety Disorder (GAD)-7 (38)

UNICEF, United Nations International Children's Emergency Fund; WG, Washington Group; CFM, Child Functioning Module; RAM, Rapid Assessment Musculoskeletal Impairment; CDST, Communication Disability Screening Tool; AP, assistive product; rATA, Rapid Assistive Technology Assessment; VA, visual acuity; RAAB, Rapid Assessment of Avoidable Blindness; RAHL, Rapid Assessment of Hearing Loss.

^a^ The WG question set screens for functional difficulties, asking about activity limitations in specific domains.

^b^ AP use access questions: problems, source, payment, distance travelled, satisfaction/dissatisfaction, assessment and training, repair, maintenance and follow-up services, suitability in the home and surroundings, and the extent to which it helps in daily life and public space.

^c^ Pilot test 2 only.

^d^ Methods need to be further tested/refined. Otoacoustic Emissions (OAE) testing was not available for the pilot.

##### 2.4.3.2 Impairment screening

In pilot 2, all participants aged 5 years and older underwent visual acuity testing. This was done to assess whether including visual acuity screening in stage 1, using a mobile VA test integrated into mFNAT, was feasible in terms of time requirements and the capacity of interviewers. Peek Acuity, a validated Android-deployed visual acuity test (18), was used for participants aged 5 years and older. Presenting vision (i.e., with glasses if participants usually wear them) was measured in each eye. Participants aged 40 years and older also had their near vision tested using the Peek Near Vision test, with a binary cut-off of N6 to determine the need for near-vision glasses (19).

##### 2.4.3.3 AP awareness and use

All participants were asked about AP awareness (20, 21) and AP use and access using questions from the WHO rATA (22). Digital images of AP, developed by the WHO, were shown to participants using the application, and/or descriptions of the AP were read aloud.

#### 2.4.4 Second-Stage assessments

Participants “screening positive” in stage one (see Table 2 for the screening criteria) underwent second-stage assessments (see Table 3) in the corresponding domain, conducted by the relevant clinician, to identify service/AP needs. The second-stage assessment methodology is detailed in Table 3. The number of people “screening positive” in each domain was monitored during the pilot testing to assess feasibility and identify whether adjustments to the screening criteria were needed.

**Table 2:** Seven functional domain two-stage “screening positive” criteria used in pilot test 2.

Functional domain	Two-stage “screening positive” criteria		
	2–4 years old	5–17 years old	18 years and older
Vision Distance Near	UNICEF/WG CFM wears glasses OR some/worse difficulty seeing –	Presenting VA < 6/12 in either eye using Peek Acuity OR test not possible and reports some/worse difficulty vision –	Presenting VA < 6/12 in either eye using Peek Acuity OR test not possible and reports some/worse difficulty vision 40 years and older: Peek Near Vision cannot see N6 or test not possible and reports some/worse difficulty vision
Hearing	UNICEF/WG CFM uses a hearing aid OR some/worse difficulty hearing	UNICEF/WG CFM uses a hearing aid OR some/worse difficulty hearing	WGES uses a hearing aid OR some/worse difficulty hearing
Mobility	RAM six screening questions “yes” to difficulty/pain using limbs or body and use of AP and duration ≥1 month		
Communication	CDST some/worse difficulty speech, communication, and understanding		
Cognition	UNICEF/WG CFM some/worse difficulty understanding, learning, playing, and behaviour	UNICEF/WG CFM some/worse difficulty learning, remembering, concentrating, accepting changes, controlling behaviour, and making friends	WGES some/worse difficulty remembering and/or concentrating
Self-care General Incontinence	– –	UNICEF/WG CFM a lot/worse difficulty with self-care Some/worse difficulty bowel/bladder function OR any recent changes OR “Yes” to numbness or tingling or loss of sensation between legs or buttock region or lower back	WGES some/worse difficulty with self-care OR most days/worse pain and somewhere between a little and a lot/more level pain OR most days/worse fatigue and most of day/worse length and somewhere between a little and a lot/ more level
Mental health	–	UNICEF/WG CFM a lot/worse depression and anxiety	WGES weekly/worse difficulty anxiety and somewhere between a little and a lot/more level OR weekly/worse difficulty depression and somewhere between a little and a lot/more level

UNICEF, United Nations International Children's Emergency Fund; WG, Washington Group; CFM, Child Functioning Module; VA, visual acuity; ES, Extended Set of Questions; RAM, Rapid Assessment Musculoskeletal Impairment; CDST, Communication Disability Screening Tool.

**Table 3:** FNAT pilot test 2 stage-two assessment tool methodology by functional domain module.

FNAT module	Tool	Stage	Age	Method	Severity thresholds	Equipment^a^
Vision		Screen	2–3 years	Fix and follow^b^	Cannot fix and follow.	Tablet
			3–4 years	Finger counting^b^	Cannot count fingers.	Tablet
	Adapted Rapid Assessment of Avoidable Blindness (RAAB) (31) using Peek Acuity (18) and Peek Near Vision (19, 23)		≥ 5 years	Distance vision: Peek Acuity mobile application visual acuity (VA) test with a pinhole to assess uncorrected, corrected, and presenting	Presenting VA in the better eye i) No impairment: ≥ 6/12; ii) mild: <6/12 but ≥ 6/18; iii) moderate: <6/18 but ≥ 6/60; iv) severe: <6/60 but ≥ 3/60; v) profound (blind): VA <3/60	Tablet; Peek Acuity and Near Vision mobile applications; pinhole
			≥ 40 yrs.	Near vision: Peek Near Vision screen	Cannot see using N6 threshold	
		Impairment assessment	≥ 2 years	Eye examination using an ophthalmoscope to assess the cause of distance VI following the WHO principle of “easiest to treat”(45)		Ophthalmoscope
Hearing		Screen	2–4 years	Otoacoustic Emissions (OAE) test or reported question asked to a relative/neighbour^b^	Fail OAE in both ears or a family member reports hearing loss in one or both ears	Tablet; OAE machine, if available
	Adapted Rapid Assessment of Hearing Loss [RAHL] (32) using hearTest (33)		≥ 5 years	hearTest mobile application pure tone audiometry (PTA) test at 0.5, 1, 2, and 4 kHz in each ear	Hearing loss in the better ear Children (5–17) and adult (18 years and older): i) no impairment: <20 dB; ii) mild: 20 dB to <35 dB; iii) moderate: 35 dB and < 50 dB; iv) moderate-to-severe ≥50 dB and < 65 dB; v) severe: 65 dB and < 80 dB; vi) profound/complete: ≥80 dB	Tablet; hearTest mobile application and calibrated headset
		Impairment assessment	≥2 years	Questionnaire on clinical history and risk factors. Otoscopy examination if HI to assess ear disease and assign a probable cause as conductive, sensorineural, or mixed^c^		Otoscope
Mobility	Updated Rapid Assessment of Musculoskeletal Impairment (RAM) (version 2.0) (26, 27)	Screen	≥2 years	Six updated RAM screening questions.	Screens positive if answers “yes” to at least one of six screening questions and if >1 month or permanent	Tablet
		Impairment assessment	≥2 years	If screens positive, perform a complete assessment including observation of activities, assignment of a case definition using the case severity card and up to three diagnoses	MSI case severity card definitions: i) no impairment; ii) mild; iii) moderate; iv) severe	Tablet; 11-m rope; plastic cup; plastic bowl, coin
Communication	Communication Disability Screening Tool (CDST) (28, 29)	Screen	≥2 years	Remaining two screening questions about the impact and nature of communication disability	Impact scale: i) no; ii) a little; iii) a lot	Tablet
Cognition	UNICEF/WG CFM cognition and behaviour questions (25)	Screen	5–17 years	Six questions asking about difficulty with learning, remembering, concentrating, accepting changes, controlling behaviour, or socialising	Functional difficulty of activity limitation: i) no difficulty; ii) some difficulty; iii) a lot of difficulty; iv) cannot do	Tablet
	WGES remembering and/or concentrating (25)	Screen	18–59 years	One question asking about difficulty with remembering and/or concentrating	Functional difficulty of activity limitation: i) no difficulty; ii) some difficulty; iii) a lot of difficulty; iv) cannot do	
	Modified Oxford Cogntive Screen-Plus (OCS-Plus) (34, 35)	Screen	≥60 years	Subset of 14 tasks in eight cognition domains screening for cognitive impairment.	Cognition age-graded severity score: i) spared; ii) impaired.	Tablet with OCS-Plus mobile application
Self-care	UNICEF/WG CFM self-care (25)	Screen	5–17 years	One question asking about difficulty with self-care activities	Functional difficulty of activity limitation: i) no difficulty; ii) some difficulty; iii) a lot of difficulty; iv) cannot do	Tablet
	Adapted Vanuatu Women, Water and Disability Study six screening questions (30)	Screen	5–17 years	Four questions about any difficulties and recent changes with bowel and bladder functions, and numbness/ tingling in lower body areas	Specific responses vary	
		Screen	≥18 years	Six questions about the frequency and amount of urine and bowel control problems	Specific responses vary	
	Barthels Index (BI) (36)	Screen	≥18 years	Ten questions asking about self-care activities of daily living, including feeding, bathing, grooming, dressing, and toileting activities	Dependency composite score: i) none if the BI total score is 100; ii) slight if the BI total score is 91–99; iii) moderate if the BI total score is 61–90; iv) severe if the BI total score is 21–60; v) total dependency if the BI total score is 0–20	
Mental health	UNICEF/WG CFM depression and anxiety (25)	Screen	5–17 years	Two questions asking about the frequency of feelings of depression and anxiety	Frequency scale: i) daily; ii) weekly; iii) monthly; iv) a few times a year; v) never	Tablet.
	Patient Health Questionnaire (PHQ)-9 (37)	Screen	≥18 years	Ten questions screening for depression and functioning	Severity composite score: i) no impairment if the PHQ-9 total score is 0–4; ii) mild if the PHQ-9 total score is 5 to <10; iii) moderate if the PHQ-9 total score is 10 to to <15; iv) moderately severe if PHQ-9 total score is 15 to <20; v) severe if PHQ-9 total score is >20.	
	Generalised Anxiety Disorder (GAD)-7 (38, 39)	Screen	≥18 years	Eight questions screening for anxiety and functioning	Severity composite score: i) no impairment if the GAD-7 total score is 0–4; ii) mild if the GAD-7 total score is 5 to <10; iii) moderate if the GAD-7 total score is 10 to <15; iv) severe if the GAD-7 total score is ≥15	

VI, vision impairment; HI, hearing impairment; MSI, musculoskeletal impairment; UNICEF, United Nations International Children's Emergency Fund; WG, Washington Group; CFM, Child Functioning Module; ES, Extended Set of Questions.

^a^ All tablets have the mFNAT application.

^b^ Methods need to be tested/refined further. Otoacoustic Emissions (OAE) testing was not available for the pilot.

^c^ Hearing loss (HL) definitions: Conductive hearing loss=HL and “abnormal” ear examination, indicating outer/middle ear condition and recorded as needing medical/surgical services; sensorineural hearing loss=HL plus “normal” ear examination and recorded as needing functional needs assessment; mixed hearing loss= conductive and sensorineural HL and recorded as needing functional needs assessment.

**Figure 1:**
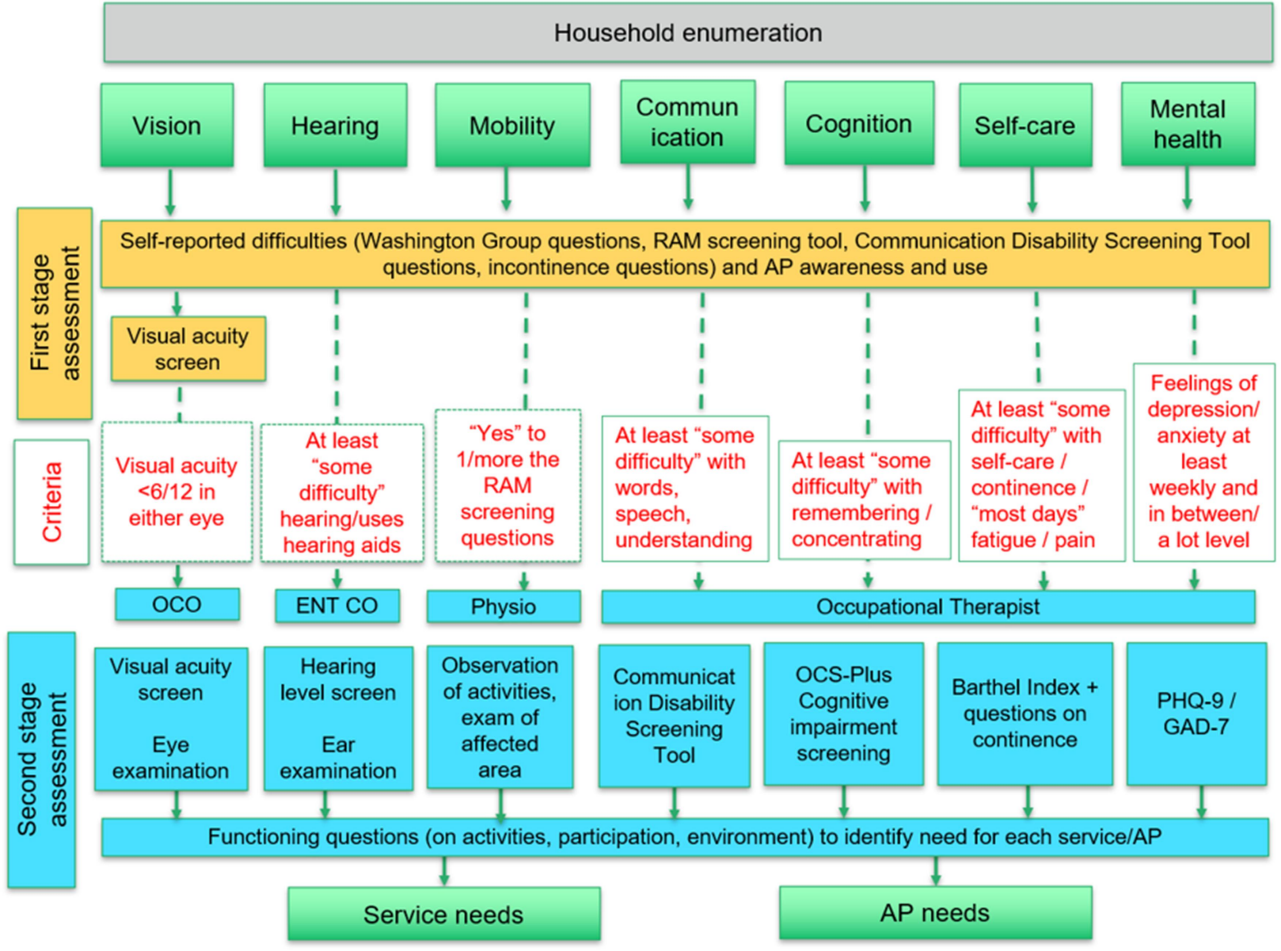
Overview of data procedures of the Functional Needs Assessment Tool (FNAT) for the pilot test in Uganda. RAM, Rapid Assessment of Musculoskeletal Impairment; AP, assistive product; OCO, ophthalmic clinical officer; ENT CO, Ear, Nose and Throat clinical officer; Physio, Physiotherapist; OCS-Plus, Oxford Cognitive Screen-Plus (OCS-Plus); PHQ-9, Patient Health Questionnaire (PHQ)-9; GAD-7, Generalised Anxiety Disorder (GAD)-7.

Figure 1 provides an overview of the overall FNAT two-stage assessment process. Across each domain, the following sections were administered by the relevant clinician to identify service/AP needs.

##### 2.4.4.1 Self-reported service use

Participants were asked about previous domain-specific service use from a list of services (see Table 4).

**Table 4:** List of service use and need indicators clinically assessed in each FNAT functional domain.^a^

List of service use indicators	Service need indicators assessed in each domain							Total domains assessing specific service type
	Vision	Hearing	Mobility	Communication	Cognition	Self-care	Mental health	
Surgery	✓	✓	✓					**3**
Medication	✓	✓	✓	✓	✓	✓	✓	**7**
Eye examination	✓							**1**
Low vision service	✓							**1**
Vision rehabilitation	✓							**1**
Optometrist/refractionist	✓							**1**
Ear examination		✓						**1**
Hearing test		✓						**1**
Hearing examination				✓				**1**
Hearing rehabilitation		✓						**1**
Physiotherapy			✓			✓		**2**
Occupational therapy	✓	✓	✓	✓	✓	✓	✓	**7**
Speech therapy		✓		✓				**2**
Prosthetics and orthotics services			✓			✓		**2**
Counselling/psychosocial support		✓		✓	✓		✓	**4**
Information on exercises without ongoing rehabilitation			✓	✓				**2**
Other rehabilitation	✓	✓	✓	✓	✓	✓	✓	**7**
Environmental modification	✓		✓	✓				**3**
Traditional medicine (use only)								**0**
Total service needs assessed in each domain	9	9	8	8	8	5	4	

a If the box is shaded with a tick mark, the service need indicator is assessed in the functional domain. If the box is empty, the service need indicator is not assessed in the functional domain. The red text indicates types of services added to the service list during training and pilot testing.

##### 2.4.4.2 Functional assessment questions

Participants identified as likely to benefit from rehabilitation services/AP were asked a set of functional needs assessment questions to identify the specific services/AP needed [note: refer to the first development paper (10) for more details]. Clinicians used this information to decide whether each service/AP should be recommended. They also recorded their clinical reasoning for this decision, which will be used to further develop/refine the algorithms in the future.

##### 2.4.4.3 Service and AP need referrals and barriers

The clinician’s assessment of service/AP need was used as the “gold standard.” The lists of services and AP (see Tables 4, 5) were adapted for local relevance based on discussions with clinicians, service providers, and the study advisory group. Participants identified as having an unmet need for any service and/or AP were asked about the reasons for this, i.e., “access barriers.”

**Table 5:** List of 26 assistive product use and need indicators clinically assessed in each FNAT functional domain.^a^

List of application use indicators	Assistive product (AP) need indicators assessed in each domain							Total domains assessing specific application type
	Vision	Hearing	Mobility	Communication	Cognition	Self-care	Mental health	
Mobile phone/smart PDA/tablet	✓	✓	✓	✓	✓	✓	✓	**7**
Long-distance glasses	✓							**1**
Reading glasses	✓							**1**
Low vision glasses	✓							**1**
Magnifying glasses, telescopes	✓							**1**
White cane	✓							**1**
Pill organisers	✓				✓			**2**
Audio players	✓			✓	✓			**3**
Talking and touching watches	✓				✓			**2**
Alarm signallers		✓						**1**
Hearing aids		✓						**1**
Walking aids			✓					**1**
Crutches			✓					**1**
Stick, cane, tripod, and quadripod			✓					**1**
Walking frame or rollator			✓					**1**
Wheelchairs			✓					**1**
Prosthetics			✓					**1**
Orthoses			✓					**1**
Protective footwear			✓					**1**
Toilet chair/commode			✓			✓		**2**
Shower/bath chair			✓			✓		**2**
Grab bars	✓		✓			✓		**3**
Ramps			✓			✓		**2**
Continence products						✓		**1**
Whiteboards- simple memory supports					✓			**1**
Communication boards or books				✓	✓			**2**
Total AP needs assessed in each domain	10	3	13	3	6	6	1	

^a^ If the box is shaded with a tick mark, the AP need indicator is assessed in the functional domain. If the box is empty, the AP need indicator is not assessed in the functional domain. AP = assistive product/s.

### 2.5 Tool translation

The FNAT modules were in English and translated into Luganda, the local national language. Existing Luganda translations of study tools were available and used for the Washington Group questions, GAD-7, and PHQ-9. The remaining questionnaires and forms were forward- and back-translated by MRC Uganda translation and survey staff to assess accuracy and equivalence. The questionnaire translations were initially tested and adapted during clinic-based testing with 2–5 participants per domain and then throughout pilot 1 and 2. In addition, the items and illustrations used in the Oxford Cognitive Screen-Plus (OCS-Plus) were adapted to the local context and redrawn when required, in collaboration with the Oxford development team, an illustrator, and members of the Uganda coordination team (AB, GT). If a participant did not speak Luganda or English, the survey team worked with a local community member and recorded this in the proxy and interview notes sections.

### 2.6 Data analysis

Feedback from the clinic, survey, and application development teams was narratively summarised. The quantitative data were descriptively analysed using Stata version 18.0 (StataCorp LP, College Station, TX, USA).

### 2.7 Ethics

Ethical approval for this study was obtained from the following research and ethics committees: the LSHTM (Ref. 28244), the Uganda Virus Research Institute (UVRI) (Ref. GC/127/923), and the Uganda National Council for Science and Technology (UNCST) (Ref. GC/127/923).

Informed written/thumbprint consent was obtained from all adults 18 years and older. For participants aged 2–17 years or for adults unable to communicate, verbal assent was sought using a simplified information sheet, and written consent was obtained from their parents/caregivers. Children had the option to complete the questionnaire or be interviewed with a caregiver present. Relevant services available within the district were identified before the survey, and people requiring referrals were provided with information through a detailed referral information leaflet. All participants received a 1 kg bar of soap, in accordance with standard practice for research in this setting.

## 3 Results

Pilot test 1, conducted in six clusters, included 305 participants (52% girls) aged 2 years and older. Pilot test 2 was conducted in 12 clusters and included 550 participants (57% girls) aged 2 years and older. Below, we describe the findings and how these were iteratively used to refine/update the methodology and the mFNAT platform throughout the pilot testing process.

### 3.1 Questionnaire updates/revision (Obj. 1)

Revisions included changes to the wording of questions to more accurately reflect the intended meaning, as well as edits to the lists of referral services and AP to better align with the local context. For example, in the self-care module functional assessment section, additional questions about the location and type of participants’ rural home bathing environments were added to improve the OTs' assessment of the need for shower/bath chairs. In addition, in the functional assessment section across all domain modules, the following activities were added to a list of tasks that were difficult to perform due to functional limitations: fetching water, participating in social activities (e.g., going to church, playing football), reading (e.g., books, the Bible, newspapers), and gardening/digging. Furthermore, traditional medicine was added as a service type in the service use section (see Table 4).

### 3.2 mFNAT tablet-based mobile application and web-based platform functionality (Obj. 2)

The training and pilot studies identified important learnings for the bespoke mFNAT tablet-based mobile application and web-based platform, which were implemented throughout the study. The following areas were improved/refined: user login features; assignment of permissions according to the different data collector roles (supervisors, trainers, fieldworkers, and clinicians); implementation of programming for the module and section filter criteria and questionnaire skips; and customised offline/online data synchronisation features between the tablets and the server. Adaptations were made to account for variable/slower WiFi connectivity and to limit local data storage on each tablet. The Luganda translations of the FNAT module could not be loaded to the application due to a technical issue, so the survey was administered through real-time translation after additional translation review/training. The one exception was the translated Luganda version of the OCS-Plus application, which launched successfully and was used in pilot 2. Due to technical difficulties, the time-stamped data were accessible only to the development team and were therefore not included in our findings.

### 3.3 Overall survey feasibility (Obj. 3)

In both pilots, the two-stage assessment conducted over 2 days was feasible, and follow-up rates were high. Based on learnings from pilot 1, both teams found it beneficial in pilot 2 to contact the village chairman/person in charge to confirm survey arrangements before arriving on the day. In addition, it was important to accurately record at least two phone numbers/contact methods for each household member to facilitate scheduling of day 2 clinician assessments or revisits. If a participant screened positive in more than one functional domain, the different clinicians coordinated the timing of their visits to minimise clinician wait time and allow participants to take breaks, thereby reducing the response burden.

The pilot studies highlighted the need to adjust the screening criteria for mental health, self-care, and cognition domains to improve feasibility. It was not feasible for the OTs to administer their assessments to all participants using the original criteria, and many participants did not require referrals. For example, in pilot 1, the vast majority of participants reported feeling tired/exhausted on “some days”; therefore, we revised the “screening-positive” criteria to “most days” in pilot 2.

### 3.4 Cluster size (Obj. 4)

The pilots provided important information about the number of participants that could be visited in a day. For participants aged 2 years and older, a cluster size of 45 participants/cluster was feasible. Across the seven domains, 5.1%–25.1% of participants aged 2 years and older screened positive for each functional domain second-stage assessment module (using the updated screen criteria). Each enumerator/interviewer interviewed approximately 15 participants/day, while each clinician assessed approximately 8–15 participants/day (see Table 6 for details). Before a full survey, the self-care and cognition screening criteria require further review/refinement due to the high number of participants screening positive and the OT assessments determining no need for services/AP.

**Table 6:** Number of pilot test two participants “screening positive” in each of the seven FNAT functional domains in two different age groups.

Survey age groups	Module screening criteria pilot 2							
	Positive							Negative
	Vision *N* (%)	Hearing *N* (%)	Mobility *N* (%)	Communication *N* (%)	Cognition^a^ *N* (%)	Self-care^a^ *N* (%)	Mental health *N* (%)	No domains *N* (%)
2 years and older (*n* = 550)	111 (20.2)	44 (8.0)	92 (16.7)	30 (5.5)	138 (25.1)	125 (22.7)	28 (5.1)	299 (54.4)
40 years and older (*n* = 134)	84 (62.7)	23 (17.2)	63 (47.0)	10 (7.5)	62 (46.3)	76 (56.7)	16 (11.9)	19 (14.2)

^a^ Cognition and self-care “screening positive” criteria require further review and edits prior to the full study due to high number of participants screening positive.

The data were also reviewed to determine the cluster size if the surveys were limited to participants aged 40 years and older, based on feasibility/resourcing (see Table 6). The proportion of participants screening positive in this age group was higher (7.5%–62.7% across the seven domains); therefore, smaller cluster sizes would be required to allow enough time for clinicians to complete their assessments. In addition, survey teams would need to complete more visits to separate houses to identify only older participants (24% of pilot 2 participants were aged 40 years and older), and vision screening criteria could be reviewed. A maximum cluster size of 30 participants/cluster is recommended, and this approach requires field-testing.

### 3.5 Vision and hearing screening for all participants (Obj. 5)

We found that it was feasible for interviewers to conduct distance and near visual acuity testing as part of stage one screening for all participants aged 5 years and older using Peek Vision (23). For participants who screened positive (i.e., VA < 6/12 in either eye, see Table 2), the OCO repeated the visual acuity screening using Peek Vision (23) before commencing the eye examination (see Table 3). Due to limited hearTest tablet resources, it was not feasible to complete hearing screening for all participants during the pilot; however, this could be explored in future studies if alternative freely available hearing software is identified.

## 4 Discussion

FNAT is a novel multi-domain, population-based survey tool designed to estimate functional difficulties/impairments and the need for services and AP by integrating self-report, clinical impairment and functional assessment methodologies. Using the bespoke mFNAT modular tablet-based mobile application and web-based platform, pilot data were collected from the district population of Kalungu, Uganda.

The pilot studies generated important learnings and highlighted areas for future development prior to administering a full survey. Updates and improvements to both the survey method and the mFNAT platform content and programming features were identified, and a full field survey is required to test these further.

Our previous research found that, using a two-stage approach, the WG questions identify people with service/AP intervention needs with only moderate sensitivity (24). We therefore included additional stage-one tools in FNAT to improve identification. However, this area deserves further attention, including the review and testing of alternative screening tools. For example, assessing the feasibility of including rapid hearing screening and/or additional self-report questions may be beneficial. Also, visual acuity data from first-stage (interviewer) and second-stage (clinician) assessments could be compared to assess agreement. In addition, the pilots provided important insights into the “screening positive” criteria and the feasibility of the two-stage data collection approach. For example, OTs reported a high number of “false positives” in the mental health domain during pilot 1, leading to further restriction of the WG question criteria in pilot 2. The pilot studies also highlighted the need to further review the criteria for the self-care and cognition domains prior to conducting a full field survey. It should also be noted that pilot 2 questionnaires will be subject to modification as the tool continues to develop.

This multi-domain survey tool was feasible to administer in a rural district sample population using digital data collection platforms to holistically assess functional difficulties and the need for services/AP. However, the tool is complex, and the assessment modules are lengthy. Further analysis using a larger dataset is needed to identify any redundant questions and to assess the added value of combining different assessment methods in assigning intervention needs. Also, the tool is relatively resource-intensive, as it utilises four different clinical cadres. Further studies are needed to explore the feasibility and accuracy of conducting assessments with fewer or a single type of clinician (e.g., physiatrists) to reduce costs and facilitate wider implementation. Finally, the development and pilot phases have further reinforced the large scope for ongoing FNAT development within individual domains and across domains. Reviewing full-survey data and methods will be important for informing ongoing development and any revisions aimed at simplifying the tool.

### 4.1 Limitations

This was the first time FNAT was used in a study setting involving multiple data collectors using the bespoke mFNAT software. The pilot studies highlighted three key limitations and challenges. First, pilot 1 identified issues related to access to and use of the mFNAT platforms, while pilot 2 identified issues with the functionality of the tablet-based mobile application and web-based platform during the two-stage assessment. Improvements to the software were implemented before/after each pilot, and any impacts on the study and timeline were proactively addressed and mitigated through (i) regular staff feedback sessions and (ii) additional application development and survey resources. Second, the recruitment of multidisciplinary teams was challenging due to the rural location, the short duration of the survey position, and the limited availability of trained clinical personnel within the country, such as OTs and physiotherapists. Therefore, adjustments were made to reduce the number of required survey teams to two, and additional training sessions were conducted. Third, the Luganda translations of the FNAT module could not be uploaded to the application due to a technical issue, so the survey was administered in real-time translation after additional translation training. Despite this training, there remains a possibility that some interviewers may have translated differently in real time. It will be important to review language option functionality when developing the mFNAT platform in the future.

## 5 Conclusion

Overall, FNAT, a new multi-domain survey tool for estimating the prevalence of functional difficulties/impairments and the need for services/AP, was found to be feasible. The pilot testing of the survey and the tablet-based mobile application and web-based platforms identified key insights and areas for future development. Conducting full surveys in different settings will be important to inform the tool's ongoing development and to demonstrate how FNAT can be adapted or scaled for use in other country contexts, such as for workforce planning and AP procurement. FNAT data have strong potential to inform policy, programming, and planning of health and rehabilitation services and AP at national levels. The field-test study is presented in the final paper in this series.

## Data Availability

The raw data supporting the conclusions of this article will be made available by the authors, without undue reservation.

## FNAT team

Suzanne Adkins, Oluwarantimi Atijosan-Ayodele, Banan Enock, Annet Birabwa, Dorothy Boggs, Sahan Bulathwela, Allan Buyinza, Festo Bwambale, Catherine de Cates, Thomas Doggett, Allen Foster, Cathy Holloway, Frank Kakuru, Hannah Kuper, Samuel Kyeyune, Joseph Mugisha, Caroline Peace Najjuma, Justine Nalweyiso, Suzan Sharon Nantumbwe, Priscilla Tracy Ngozi, Joan Nkamushaba, Sarah Polack, Emmanuel Taika, Mary Theresa Tuhaise, Grace Tulinaomubezi, Wilson Tumwine, and Stella Twinamatsiko.
