# Supplementary material for: Functional Needs Assessment Tool for estimating population-level functional difficulties and the need for services and assistive products: pilot testing in Western Uganda
Source: Front Rehabil Sci. 2026 Jul 21;7:1766661. doi: 10.3389/fresc.2026.1766661 (PMC13435221; doi:10.3389/fresc.2026.1766661)
Supplement: Supplementary file 1 [file Table1.docx]

**Supplementary Table 1. FNAT terminology and definitions used in the paper**

| **TERM** | **DEFINITION** |
| --- | --- |
| Assistive products | Any external product (including devices, equipment, instruments or software), especially produced or generally available, the primary purpose of which is to maintain or improve an individual’s functioning and independence, and thereby promote their well-being. AP are also used to prevent impairments and secondary health conditions.(3.4) |
| Assistive technology | The application of organized knowledge and skills related to assistive products, including systems and services.(3,4) |
| Functioning | An umbrella term in the International Classification of Functioning, Disability and Health (ICF) for body functions, body structures, activities, and participation. It denotes the positive aspects of the interaction between an individual (with a health condition) and that individual’s contextual factors (environmental and personal factors).(4) |
| Rehabilitation | A set of interventions designed to optimise functioning and reduce disability in individuals with health conditions in interaction with their environment.(5) |
